# Factors contributing to the mental health outcomes of carers during the transition of their family member to residential aged care: a systematic search and narrative review

**DOI:** 10.1186/s12877-022-03105-4

**Published:** 2022-05-17

**Authors:** Vera Camões-Costa, Jayasree Loganathan, Chris Barton, Samantha Chakraborty, Alana Hewitt, Xiaoping Lin, Bianca Brijnath

**Affiliations:** 1 Department of General Practice, Monash University, Building 1, 270 Ferntree Gully Road, Notting Hill, VIC 3168 Australia; 2 Department of Occupational Therapy, Monash University, Frankston, Victoria Australia; 3 Department of Epidemiology and Preventive Medicine, Monash University, Melbourne, Victoria Australia; 4 National Ageing Research Institute Ltd, Parkville, Australia; 5 School of Occupational Therapy, Social Work and Speech Pathology, Curtin University, Perth, Australia

**Keywords:** Residential aged care, Nursing homes, Long-term care, Mental well-being, Family carers, Review, risk factors

## Abstract

**Objectives:**

The transition of an older family member into a residential aged care facility (RACF) is often challenging for both the person being admitted and their family carer. This review aimed to identify the protective and contributing factors to adverse mental health outcomes among family carers following the decision to move a family member to a RACF.

**Method:**

A search of CINAHL, PubMed and PsycINFO was conducted for empirical papers published in English between 2004 and 2019, exploring the mental health or quality of life (QoL) of family carers of those recently admitted, or considering admission, to a RACF. Articles were reviewed by two authors for inclusion.

**Results:**

Twenty-three studies met the inclusion criteria. Pre-existing depressive symptoms and poor subjective health were related to adverse mental health outcomes following admission. Information from the facility, support to change roles, and factors related to carer’s health and demographics, were associated with changes in the mental health outcomes of carers during the transition of their relative to a RACF. Key protective factors of carer’s mental health outcomes following the transition of their relative to a RACF are flow and transparency of information between carer and the facility staff, and staff efforts to involve carers in providing emotional support to their relative, in monitoring care, and advocating for their quality of life.

**Conclusion:**

There is evidence to suggest factors such lack of flow and transparency of information between carer and the facility staff may predispose carers to poor mental health and QoL following the transition of a relative to a RACF. Key protective factors of carer’s mental health following admission are staff efforts to involve carers in providing emotional support to their relative, in monitoring care, and advocating for their quality of life. This review also indicates that the combination of factors that puts family carers more at risk of poor mental health and lower quality of life throughout the transition period. Policy and practice should follow recommendations that consider a combination of the above factors when addressing the needs of family carers before and after admission of an older person to RACF.

**Supplementary Information:**

The online version contains supplementary material available at 10.1186/s12877-022-03105-4.

## Background

Family carers play a crucial role in the life of older people. The physical and mental functional capacity of a family carer often determines how long the older person can continue to age at home [1]. International ageing policies and service planning schemes promote independence, active choice, and social inclusion for older people. Supporting people’s preference to live at home for as long as possible is the focus of many governmental initiatives both nationally and internationally [2, 3].

In Australia, policy makers are currently challenged by the growing demand for home care packages given the increasing number of people aged over 65 [4]. In 2018, 1.3 million older Australians living at home needed some assistance with everyday activities, and of these, over one-third (34%) did not have their needs fully met [4]. Two thirds of people who began using aged care services were admitted into residential care. Family carers are instrumental in helping older adults age in their own homes, and the care they provide often replaces or complements formal (paid) services to meet the needs and preferences of older people. The well-being, mental health, and quality of life of carers is challenged by the transition to residential care (e.g., [5–7]). Carer’s symptoms of depression and anxiety were not found to improve significantly with admission to residential care, particularly for spouses and those who were less satisfied with residents care or who visited more often [8]. In the same study, carers use of antidepressants before admission had also not changed with admission, rather a significant increase in the carer’s use of anxiolytic (14.6 to 19%) was reported, and nearly 50% were at risk of clinical depression after placement [8]. The aim of the current review, however, is to identify the protective and contributing factors to adverse mental health outcomes of carers when an older family member transitions to a RACF. In 2017–18, the majority of people entering permanent residential aged care were aged 85 years and over (59%). By extension, family carers (whether spouses, siblings, or adult-children/in-laws) are often older adults themselves [9] with associated age-related conditions. With women comprising 70 % of informal carers [10], the increase in women’s work force participation, social demands and expectations often competes with their ability to provide care for an older family member [9]. Juggling competing responsibilities, often has negative effects on the health and wellbeing of this so called ‘sandwich generation’ [9], where individuals care for those in the generations above and below them. For this reason, the health and wellbeing of the family carer (hereafter carer) is often fragile and compromised by the time an older person is admitted to residential aged care.

Understanding the effects of long-term care placement on the mental health of family carers, warrants a review of the literature. Although reviews on the wellbeing of informal carers of people with dementia transitioning to residential care have been conducted (e.g., [5–7])., this review aims to expand on this, with a focus on the wellbeing of all carers of older people regardless of care recipients’ health condition.

Many newly admitted residents and their carers feel the decision regarding entry into residential care is rushed and overwhelming [7]. Admission often follows a traumatic event, such as a fall or hospitalisation, illness, disability, bereavement, an emergency, the changing needs of their carer, or because it is no longer possible to manage at home. The wellbeing and quality of life (QoL) of carers of older people is highly challenged with the transition of their older family member to a residential aged-care facility (RACF) [11]. A RACF in Australia is a facility where older people reside, when they can no longer live at home and need ongoing help with everyday tasks or health care. This is also known as a nursing home, aged care home, long-term care, or skilled nursing facility in the international literature.

Previous research has shown that the pre-admission experiences may have an impact on how carers experience the transition [12, 13]. In addition, a difficult pre-admission experience, such as feeling unsupported in their decision making, feeling pressured by professionals towards admission, or not having the efforts involved in their caring role validated by others, may also hinder the effectiveness of support systems for carers provided in the post-admission stage [13–17].

After admission to a RACF, it is not unusual for carers to experience a loss of purpose in life [18], as their role changes in many ways and often leaving carers to establish a new role [5, 19]. The new role is often one of advocating for good quality of care [20] and respect for their relative’s dignity [16, 21], linking the resident to the world external to the facility [20], or assisting their family member integrate into their new environment by contributing to the RACF community [20].

To date, studies indicate that the experience of carers during the transition of their loved one to RACF is varied [15, 18, 22]. Carers may experience significant relief knowing that their family members now receive quality care from trained staff instead of themselves [16, 18, 21, 23], and that the struggle of finding a RACF is over [17]. Other carers may experience ongoing poor mental health [24], and a range of negative emotions during the transition of older people in a RACF [6, 25].

The well-being, mental health, and quality of life of carers is challenged by this transition, with admission often impacting negatively on carer anxiety, depression, burden, and loneliness [5–8, 26]. While the experience of carers has been widely explored [6], the characteristics that make carers more susceptible to ongoing burden and poor mental health, are less defined. Identifying the factors that increase carers risk of poor mental health outcomes after admission, is important to help target timely support for those most vulnerable. In addition, such targeted support may also impact the mental health outcomes and adjustment of clients entering the facility [16]. Given the exploratory and broad nature of our aim, and the variety of ways evidence on how variables related to mental health and quality of life of family carers are are collected, reported and analysed, a narrative review and synthesis methodology was considered the most appropriate approach to take. Systematic reviews are useful for answering well defined research questions with a narrow, specialised scope, but are less relevant when little is known about a topic and where a broader search and scoping of literature is initially required [27, 28].

The current review seeks to describe the range of factors contributing to poor mental health and wellbeing among family carers following the decision to move a family member to residential aged care and after admission to permanent residential care takes place.

## Methods

### Search strategy

A systematic search of peer reviewed literature indexed in electronic databases CINAHL via EBSCOhost, PubMed, and PsycInfo via OvidSp was completed using the following terms: (1) participants (i.e., carers of older people); 2) outcome (e.g. QoL, burden, mental health, wellbeing); and (3) setting (e.g. residential aged care, nursing home; admission; transition of care). With the impacts of residential care transition on family carers gaining attention over the past 15 years, literature included ranged from 2004 to 2019 [7] (see full search strategy in Supplementary File Box 1).

### Inclusion/exclusion criteria

We used PICO (population, intervention, comparison, and outcomes) as the framework to select relevant studies. We included any descriptive, correlational, or causal-comparative/quasi-experimental studies, reporting relationships between the mental wellbeing and QoL of family carers of older people and their circumstances before or after of the admission of their older relative to residential aged care. The exclusion criteria were studies examining intervention outcomes, residents’ outcomes only, conducted in non-RACF settings (e.g. retirement villages), those including paid carers, or with samples lower than two participants. Intervention studies and studies testing validity of instruments were also excluded to avoid outcome reporting bias. Review articles were excluded to avoid duplication of findings, as the reviews identified often included a number of articles papers included in the current review. However, review articles were used as a source for citation searching relevant papers.

The study selection was carried out by two reviewers independently (JL and VCC), using EndNote to assist with this process. After removing duplicates, the title and abstracts of all studies were screened for relevance and eligibility against the inclusion criteria and those meeting these criteria the full text was downloaded and assessed for study eligibility. Citation searching of references from included articles was also conducted, with no additional studies found. Screening and assessment of eligibility was conducted by JL and reviewed by VCC. Discrepancies were resolved by discussion.

### Data extraction and analysis

This review follows recommendations for conducting and reporting a review of independent syntheses of qualitative and quantitative [29], where qualitative and quantitative findings are interpreted together in the discussion. The data extracted from the studies included country in which the study was conducted; study objectives, participant characteristics; study design; outcome measures; period in the transition data was collected; and relevant findings. Investigator JL and investigator VCC conducted data extraction and critical appraisal, with full agreement achieved through discussion. The quality of the articles was appraised using the Mixed Methods Appraisal Tool (MMAT) [30], which includes specific criteria for appraisal of qualitative and quantitative research [30].

Relevant data were extracted by two authors (VCC and JL) and an iterative review of each study, using an inductive methodology for the thematic synthesis of the qualitative data [31] was undertaken. Extracted text was examined for common characteristics and relationships between variables which enabled concept mapping across both pre-admission and post-admission periods of the transition. Emerging themes were discussed and agreed between the research team members. A convergent synthesis of the quantitative results added insight to the integration of the findings [29].

## Results

**Fig. 1:**
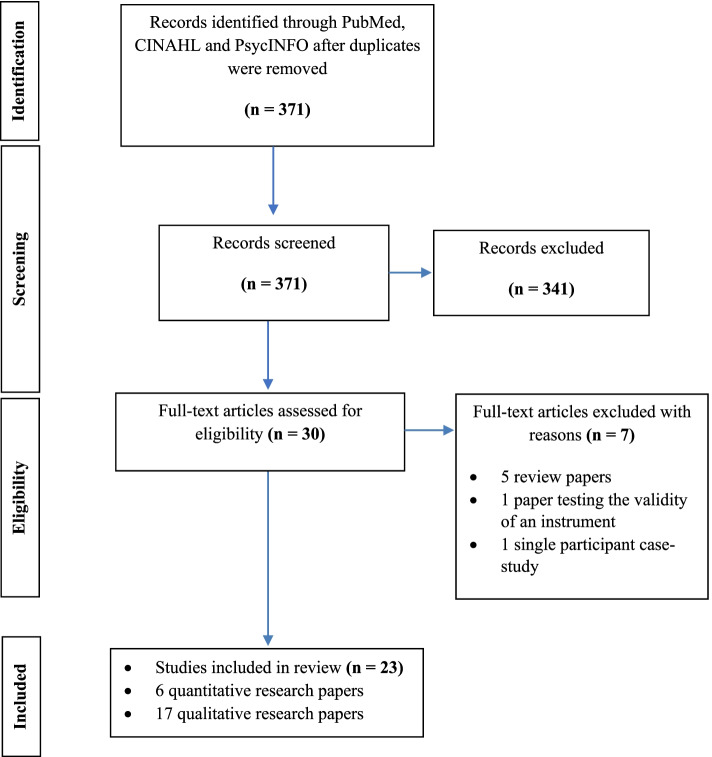
PRISMA flow diagram

From the original 371 articles identified via the databases, 341 articles were excluded as these either reported on the outcomes of interventions, or the participants included were aged care residents or paid carers. Thirty full text-articles were then assessed for eligibility. From this, 7 papers were excluded including: review papers (*n* = 5), a paper testing the validity of an instrument (*n* = 1) and a single participant case-study (*n* = 1). No study was excluded from the systematic search based on its critical appraisal (see results of quality assessment using MMAT in Supplementary File Box 2), given they were all of high enough quality. Overall, 23 studies were included in this review (Fig. 1). These studies varied in the measures used and outcomes reported, and used a mixture of qualitative and quantitative methodologies. As such, a meta-analysis of the existing evidence was not appropriate. Instead, a narrative review was conducted (Table 1 details the 23 studies included). Of all included studies, 17 had a qualitative aspect to its methodology and used interviews to gather their data [12–18, 20, 23, 32–39], and 6 reported quantitative findings [40–45].

**Table 1:** Characteristics of the 23 studies included in the review

Study Author, Year, Country	Objectives/Aim	Sample	Study Design	Outcome measured/explored
Barken & Lowndes, 2018, Canada [32]	To identify practices and conditions that promotthe dignity and respect of workers, residents, and unpaid carers	149 unpaid carers of residents in long term care	Qualitative study, using rapid ethnography	Challenges in various stages of long term residential care
Bleijlevens et al., 2015, Holland [40]	Determine how transition to RACF affects caregiver burden and health related QoL	109 carers of people with dementia	Prospective cohort study with 3 m follow up	Burden - ZBI and caregiver reaction assessment (CRA) HR QOL - EQ-5D
Bramble et al., 2009, Australia [12]	Examine the emotional challenge and new roles of carers after transition	Ten carers of people with dementia	Qualitative study with semi structured interviews	Experiences of family caregivers who have placed a relative with dementia into long term care
Crawford et al., 2015, Australia [18]	To explore caregiver experiences during transition	20 unpaid carers (between 34 and 92 years old) of people with dementia	Qualitative study with semi structured interviews	Carer roles; experience of carers; factors that affected the coping of carers
Davies & Nolan, 2006, UK [20]	Understand the contribution of family carers to life within the home	37 close relative of people admitted to a nursing home	Qualitative study with Semi structured interviews	Roles of carers in RACF
Davison et al., 2019, Australia [23]	Explore the views of residents with dementia, families of residents with dementia and facility staff on enablers and barriers to successful adjustment to RACF	38 carers of people with dementia	Qualitative study with semi- structured interviews	Experiences from the point of view of residents, their families and staff
Eika et al., 2014, Norway [16]	To understand the experiences of carers during the transition of their family members to nursing homes	Ten next-of-kin of recently admitted residents	Qualitative study with semi- structured interviews	Experience on admission day Experience in initial period after placement
Gaugler et al. 2007, USA [43]	Identify long term effects on caregivers of PwD whom move to RACF	146 caregivers of patients diagnosed with dementia, with at least 2 years of post placement data; 38 caregivers of patients diagnosed with dementia, with 5 years of post placement data	Longitudinal cohort study based on data from the caregiver stress and coping study	Stress; global well being; psychosocial resources; psychological outcome - Hopkins systems checklist (7 item scale); pre placement assessment and either 2,3,4,5 waves of assessment after placement
Gaugler et al., 2010, USA [41]	Determine if there was significant changes in burden and depressive symptoms in caregivers in the 12 months following RACF admission of their relatives with dementia; identify key predictors of burden and depression in that transition period	1116 caregivers of patients diagnosed with dementia	Cohort study with follow up of 12 months	Zarit Burden inventory; geriatric depression scale; MMSE for cognitive status; memory and behaviour problems checklist; functional impairment - judged by unmet needs of relative + number of hours of caregiving per day
Gaugler et al., 2014, USA [42]	Test prognostic tools for their usefulness in identifying dementia caregivers at risk for burden and depressive symptoms.	1610 caregivers of patients diagnosed with dementia	Retrospective longitudinal study	NHA-Burden tool; NHA-depression tool
Givens et al., 2012, USA [14]	To understand the experiences of family members with regard to the transition of their older family member to nursing home	16 carers of people with dementia average age 62 years	Qualitative cohort study with semi structured interviews	Communication with HCPs; surrogate decision making; emotional distress
Hainstock et al., 2017, Canada [15]	To understand the caregiver’s journey in transitioning their older family member into RACF	15 family carers	Qualitative study with semi structured interviews	How do family caregivers navigate and experience the transition from HC into RC for a family member?
Kallianis et al., 2017, Australia [33]	To understand the impacts on family of transition of patients from palliative care to RACF	Eight family members members of patients receiving palliative care in hospital	Qualitative study with semi-structured interviews	Concerns and barriers to transition
Kelsey et al., 2010, USA [34]	Explore how caregiver-care receiver dynamics affect the experiences of the transition	15 carers of people with dementia	Qualitative study with semi-structured interviews	Experiences of caregivers during transition to assisted living
Konietzny et al., 2018, Canada [17]	To explore informal caregivers’ experiences of transitioning an older adult into long term care	13 informal carers of residential care residents	Qualitative study with semi-structured interviews	Caregivers’ experiences before, during and after transiton
Lloyd, 2010, Canada [35]	To better understand the experiences of caregivers during a crisis placement of their family member	Six primary caregivers who had crisis placed an elderly family member in a nursing home	Qualitative study with semi-structured interviews	Decision making; emotional responses
Metzelthin et al., 2017, Holland [44]	Compare caregiver and care receiver characteristics and caregiver outcomes; study the association between positive and negative caregiver outcomes; study the effect of RACF on these associations	5197 caregivers of patients diagnosed with dementia	Cross sectional study	Characteristics: sociodemographic, health related, caregiving related outcomes: subjective burden, care related QoL
O’shea et al., 2014, Ireland [36]	To explore relatives involvement in the care of their family members in RACF	Nine primary family carers of residents	Qualitative study with semi structured interviews	Family involvement in residential care
Palacios-Ceña et al., 2019, Spain [37]	To better understand the experiences of female caregivers during the transition of their family member into RACF	20 female family caregivers between ages 18 and 60	Phenomenological qualitative study	Life experience of female; family caregivers after long-stay nursing homeadmission of their relative
Pearson et al., 2004, Australia [38]	Understanding relative’s experience of the transition	58 carers who defined themselves as a close relative of someone admitted to RACF in the past 2 years	Qualitative study with interviews	Experiences and responses that each individual had before, during and following the admission of their family member to an RACF.
Ryan & McKenna, 2013, Ireland [39]	To explore rural family carers’ experience of RACF placement of their relatives and explore factors that hindered successful transition	29 rural family carers	Qualitative study with interviews	Experiences of rural carers; factors hindering transition
Schulz et al., 2004, USA [8]	Assess the impact of placing a relative with dementia in RACF on carer wellbeing report on the transition experience and postplacement health effects in a large cohort of family caregivers of persons with dementia	180 caregivers of patients with dementia	Prospective study – 18 m duration	Caregiver depression (CES-D); caregiver anxiety (state trait inventory); use of medications for depression and anxiety
Sussman & Dupuis, 2012, Canada [13]	To determine the effects of RACF admission on relatives of residents and how interventions can improve their experiences	20 carers of people with dementia or with progressive medical conditions that had become diffi cult to manage in the community, such as chronic obstructive pulmonary disease and diabetes	Qualitative study with semi structured interviews	Families’ positive and negative experiences during each temporal phase in transitional care –the decision-making period, the waiting period, the move itself, and the post-move adjustment

*RACF* Residential aged-care facility, *QoL* Quality of life, *ZBI* Zarit Burden Interview, (CRA) HR QOL - EQ-5D – (caregiver reaction assessment) based health-related quality of life (HRQoL) EuroQol 5 Dimension questionnaire; *PwD* People with Dementia, *MMSE* Mini-mental state examination, *NHA* Nursing Home Admission, *CES-D* Center for Epidemiological Studies-Depression

The sample size in the 17 qualitative studies varied from six [35] to 149 caregivers [32] and across all articles there was a total sample size of 473 family carers from 6 countries: US, Canada, UK, Ireland, Spain, and Australia. Of the six quantitative studies, four were based in the US [8, 41–43], and two in Europe [40, 44]. Three of the US-based studies were conducted by the same group of researchers [41–43], and two of these studies reported on a secondary analysis of data from the same longitudinal study [41, 42]. One of the European studies was conducted across eight countries [12, 40]. The other European study reported a secondary analysis of data collected from 25 studies conducted in the Netherlands [44].

Thematic analysis of text extracted from the qualitative studies revealed three broad themes associated with changes in the mental health outcomes of carers during the transition of their relative to a RACF: 1) information obtained and from the facility, 2) support to change roles, and 3) factors related to carer’s health and demographics.

Mental health outcomes of carers when an older family member transitions to a RACF, and the factors associated with the mental wellbeing of carers during the transition of older family member transitions residential aged care are described in further detail below.

*Mental health outcomes for carers when an older family member transitions to a RACF The quantitative studies included in this review identified a numbers of factors contributing factors to poor mental health, poor wellbeing, and poor quality of life, as well as identified protective factors (see* Table 2*).*

**Table 2:** Summary of the factors impacting poor mental health, wellbeing, and quality of life in carers

Study Author, Year, Country	Factors impacting poor mental health, wellbeing, and quality of life in carers
Bleijlevens et al., 2015, Holland [40]	Decrease in burden found at 3-month follow-up after transition, although clinical significant burden was still present then. Decrease in psychological distress found at 3-month follow-up after transition, although no significant changes in health related QOL were reported in this group.
Gaugler et al. 2007, USA [43]	Decreases in stressors and indicators of negative mental health, such fatigue due to informal role provision, anxiety and anger, were found over time.
Gaugler et al., 2010, USA [41]	Decrease in burden found at 6-month and 12-month post-placement. Smaller decrease in depressive symptoms found at 6-month and 12-month post-placement.
Metzelthin et al., 2017, Holland [44]	While objective caregiver burden (measured by the number of caregiving hours) may decrease with admission, carer’s subjective burden remained high following transition of the care recipient to permanent care
Schulz et al., 2004, USA [8]	The use of anxiolytics increased with placement and nearly 50% of carers were at risk of clinical depression after placement. Small improvements were more commonly observed in spouses whom visited frequently and those less satisfied with the care.

Objective caregiver burden, measured by the number of caregiving hours, was found to decrease with admission [40], while carer’s subjective burden remained high [44]. A decrease in burden and stressors and indicators of negative mental health, such fatigue due to informal role provision, anxiety and anger, were found over time [43]. However, at 3-month follow up clinical significant burden was still present then [40].

After admission, nearly 50% of carers were at risk of clinical depression, and the use of anxiolytics increased [8]. A decrease in psychological distress was found at 3-month follow-up and tapered over time [41, 43]. Small improvements were more commonly observed in spouses whom visited frequently and those less satisfied with the care [8]. However, no significant changes in health related QOL were found [40].

### Factors associated with the mental wellbeing of carers when an older family member transitions to a RACF

#### Information about and from the facility

The experiences of carers before admission are likely to affect their overall confidence in the transition [13]. For example, carers less familiar with the facility were more likely to deem what happened post admission as inadequate or unacceptable [32, 39]. Having information about the facility and its reputation beforehand was helpful in reassuring and increasing carer’s confidence in the care provided at a RACF. However, the decision to move an older family member into RACF was often based on little information [14, 15]. Knowing as much as possible regarding care provided at the new facility helped carers prepare and reduced the uncertainty of how the older person would integrate [33].

Post-admission, the information provided by the facility staff on how the resident is adjusting was seen as a testament of trust and respect [32], and crucial for the experience of the carer [16, 23]. Not feeling welcome in RACF, being apprehensive or ill-informed about quality of care were adverse factors to carer’s adjustment [15, 16]. Flow and transparency of information between carer and the facility staff, and staff support of regular visits prevented loneliness in the carers, and contributed to better mental health of carers [18, 37]. Barken’s study reported the following from a participant: ‘*the more contact you have with the families even though it’s time- consuming, the more transparent you are, the more honest you are about the way things are here, you know, it’s really appreciated and the more on board families are*’.

#### Support to change roles

Many carers experienced chronic worry and burden before deciding to transition their loved ones to permanent residential aged care, or felt a sense of losing control despite their pivotal role in the transition process [17]. Many carers feel guilty regarding planning or making the decision to move an older family member to an RACF, and they may see this as a betrayal of trust, or an act of abandonment [33]. Staff efforts to build relationships with family carers, identify forms of assistance the carer might need, and include family in decision making were found to be important for a smoother transition [12]. Two studies have shown that adjustment of the family members to the transition was closely related to whether the older relative accepted the placement [13, 23], and the type of relationships family member established with staff at the facility.

At the time of admission, carers experience a great sense of loss of purpose as their role as a carer dissipates and becomes the responsibility of the care staff [18]. Being able to use their knowledge of their loved one – i.e., knowing exactly what they want and how they want it - and being supported to shift into an advocacy role for the best quality of care possible helped carers to feel part of the ‘specialist’ team caring for the resident [18, 20]. Carers became more involved in providing emotional support to their relative, monitoring care, and advocating for their quality of life [36]. Good communication between staff and family, made the transition more pleasant and carers felt more included [14, 15, 23]. A participant of Hainstock & et all (2017) explained: ‘*Now, of course, the care home people are doing all the care. I’m just sort of the emotional- a bulwark of his existence now. I’m the familiarity of the past. But as far as physical care- I think I’m a lot of mental care, but no, no, there’s no physical care going on.’* Fewer visits of carers to the facility were also linked to significantly poorer relationships with staff [36, 37].

The relationship with other family members and their support regarding the decision to move a family member to residential care also contributed to whether carers found the process positive or experienced guilt, devastation, loneliness, panic and helplessness [38].

#### Factors related to carer’s health and demographics

Spouses were more affected by the move than adult children [34]. A study found that carers who were co-residents with the older person were most affected [20]. The following quote from a daughter illustrates the emotional hardship of transitioning a parent to residential aged care: *‘Honestly, right from the beginning we knew it was happening and we knew it was coming. My sister and I were so stressed, I remember going home crying numerous times. Crying at night, the two of us, we would break down. And we would be like “what are we going to do?”*. *Very helpless, our hands were tied. Because we knew that mom can’t go home, they’re not willing to help us try and it’s just like, “here’s a list of homes; go look at them and let us know’* [17]. Adult children who have gone through the process of admitting one parent to residential aged care in the past reported feeling better prepared the next time [17]. Experience of emotional ambiguity, grief, loss of control, and guilt, were also associated with worsening of chronic conditions like diabetes, pain, glaucoma, psoriasis and thyroid issues in carers [35].

More recently, the usefulness of prognostic tools in identifying caregivers at risk for burden (defined as the negative psychosocial, economic, or physical effects of providing care to a relative) and depressive symptoms was questioned [42]. Using a sample of 1610 caregivers, this retrospective longitudinal study identified that, in instances of more than 36 months of caring, any unmet need in the carer (i.e. geriatric depression scale score above 2, burden inventory score above 9), was a pre-admission predictor for ongoing burden 6 months post-placement. Having a pre-admission geriatric depression scale score above 2, subjective poor health, being a spouse, and using services routinely (i.e., housework personal care, and adult day care services) also predicted ongoing depression in the caregivers of residential aged care recipients with dementia 6 months following admission.

Logistical regression analysis indicated that the key predictors of caregiver burden and depression were gender and kin relationship to the care recipient; women were more likely to have prolonged burden; wives and daughters were at 8 and 2 times greater risk of ongoing symptoms after placement, respectively [41]. In comparison, husbands were found to be at higher risk of persistent depression. Caregivers from Caucasian background, who are employed, who provided care for longer hours prior to admission, and have greater self-reported health needs of their own (such as health impairments or lack of social support), are more likely to suffer from persistence burden at 6 and 12 months post admission [41].

A cross sectional study compared caregiver and care recipient characteristics with caregiver outcomes [44]. Care recipients who were men, married, and had increased disability and lower perceived wellbeing, experienced increased carer burden; while women and younger carers, who co-lived with the care recipient, reported increased burden and poorer self-perceived health. However, this paper reported a secondary analysis of caregivers and care recipients data drawn from 25 different studies with variation in sampling, inclusion criteria and data collection method, which may compromise generalisability of the findings.

## Discussion

The aim of this paper is to highlight the protective and contributing factors to adverse mental health outcomes of carers during the transition of an older family member to residential aged care. Significant variation in findings reported in this review suggest some carers are more susceptible to poor mental health outcomes and QoL than others.

Carers who feel unsupported during the transition of the older person to residential care are likely to experience poorer mental health outcomes [7, 36, 42]. This is consistent with qualitative data reporting poorer mental health in carers who make sudden decisions and have less time to prepare for the transition [12] and with a previous review by Jacobson and colleagues [6].

Carers with greater unmet needs of their own, such as those unable to meet their own commitments (e.g., employed carers, those with financial difficulties, poor social lives, or declining physical health) seem to be more likely to experience greater ongoing burden, mental and physical exhaustion, and may resent their caring role. These carers also tend to prefer to visit less often. Conversely, those more socially engaged and satisfied with their carer role, who feel supported during the transition are more likely to experience greater QoL and less burden and depression and feelings of loneliness following the admission. Future research to further explore the associations above is warranted, to allow the development of targeted interventions for carers at risk.

Those who feel forced to visit regularly and provide ongoing care are at an increased risk of poorer mental health outcomes [8]. However, the reason for such increased risk is also likely to be multifactorial. Dissatisfaction with services and communication by staff has been related to increased depression in carers [7, 8, 13]. It has been proposed that carers dissatisfaction with care services may promote greater apprehension and guilt, and therefore higher visitation and caregiving, and might be an underlying cause for poor mental health of carers [8]. Additionally, the loneliness associated with being a spouse of someone admitted to a RACF often stemmed from isolation from other social connections that result from frequent visiting and caregiving [8]. Carers who feel more socially isolated (such as single carers, co-resident carers and spouses), dissatisfied with the care provided, and poorly supported during the transition exhibit greater levels of burden and negativity regarding the transition. These carers are often people whom had neglected their own social lives and other relationships during their caring role; carers with fewer social engagement activities seem to express greater feelings of loneliness and are also more likely to feel depressed [46].. Variation in the outcome of the transition for carers was also reported in a previous review by Sury and colleagues [7]. However, the studies included in that previous review provided fewer insight of what may influence the trajectory of these different outcomes.

A strength of this review, in comparison with previous reviews [5, 6],is that both quantitative and qualitative studies were included, allowing a more thorough exploration of factors impacting informal carers’ wellbeing. Previous findings are inconsistent in showing changes in carer’s burden after RACF admission of family members [5]. However, this does not mean carer’s mental health improve with the transition. Previous data also supports that some carers continue to report poor mental health outcomes post admission into RACFs. An earlier review by Afram and colleagues [5] suggested the need to consider the care-transition period a continuum, rather than adjacent stages. The findings of the current review still support the view of an integrated transition stage where pre-admission factors may influence the trajectory of post admission outcomes for carers. However, how the transition influences pre-existing depression and anxiety in carers is still unclear [8, 42].

Conflicting evidence suggests that transition to a RACF may alleviate negative emotional symptoms in some family carers, but a large proportion of these carers continue to experience ongoing depression, burden, and poor QoL. This may also be because the consequences of care such as reduced income will have a longer term impact, or because some mental health conditions are chronic rather than situational [47]. In addition, the studies included in this review involve a heterogeneous sample of aged care residents in regards to their care needs, and specifically in relation to the presence of dementia. It is not uncommon for transition to aged care to be accompanied by advancing dementia. Carers of people living with dementia experience four times worse mental health than non-carers [48]. Therefore, dementia in the care recipient in an important contributor to poor mental health in carers.

### Limitations

This review includes only articles published in peer-reviewed journals. Although it is likely that other articles exist outside of these sources, it is unlikely that these articles would be of adequate methodological quality. Studies not published in English were excluded, limiting our ability to examine relevant data from non-English speaking countries or lower-middle-income countries. In addition, this paper reported a secondary analysis of caregivers and care recipients data drawn from 25 different studies with variation in sampling, inclusion criteria and data collection method, which may compromise generalisability of the findings.

There is a lack of standardization across the literature as to what constitutes the transition period to a RACF. As such, the studies included covered various timelines from pre-admission to more than 5 years after admission, and examined carer experiences during various phases after transition. Additionally, the participants were all carers of people who are either entering or have entered residential age care, and no specific information is provided about the level of cognitive impairment or the type of care received by the residents. This caregiver population may have been caregiving for a long period of time already and not be representative of all carers. Future research should explore predictors of poor mental health outcomes in family carers of newly admitted residents. Additionally, despite referring to unmet needs in carers, the included articles did not specifically identify these. Clearly understanding unmet needs provides an avenue for intervention.

Also, we may have missed key terms in the search strategy, which may have resulted in additional key papers not being identified and included in this review, limiting the findings we are able to report. *Implications for policy and practice.*

Information regarding the combination of factors that puts family carers more at risk of poor mental health and lower quality of life throughout the transition period can inform the development of screening tools or targeted early interventions to help carers in need of additional support at specific times during the transition.

There is evidence to suggest factors such lack of flow and transparency of information between carer and the facility staff may predispose carers to poor mental health [18, 33, 37] and an overall negative experience following the transition of a relative to a RACF [16, 23, 32]. Key protective factors of carer’s mental health following admission are staff efforts to involve carers in providing emotional support to their relative, in monitoring care, and advocating for their quality of life.

This review also indicates that it may be the association of certain characteristics, which increase the subjective burden, and decrease QoL and mental health outcomes for carers. For example, spouses, carers who co-reside with the older person, with pre-existing depressive symptoms, and poor subjective health, seem to have poorer mental health outcomes over the transition period [20, 34, 41, 44].

This means that policy and practice should follow recommendations that consider a combination of the above factors when addressing the needs of family carers before and after admission of an older person to RACF. For example, specific assessment and advice tailored to individual needs may be recommended. More robust evidence based on valid and reliable mental health measures across different time points of the transition is needed. How burden, depression and quality of life in carers varies over time during the transition period (i.e, in preparation for admission, shortly after admission, or some months after admission), and which factors make carers more susceptible to ongoing poor mental health at different points of the transition, should be further explored to inform policy and practice into supporting carers of prospective and newly admitted aged care residents.

## Supplementary Information

**Additional file 1.**

**Additional file 2.**

## Data Availability

The research reported in this paper involves the use of non-identifiable data or records from previously published research.

## Abbreviations

RACF: Residential aged care facility
QoL: Quality of life

## References

[1] Committee on Family Caregiving for Older Adults; Board on Health Care Services; Health and Medicine Division; National Academies of Sciences E, and Medicine;. Family Caregiving Roles and Impacts. Schulz R EJ, editors., editor. Families Caring for an Aging America Washington (DC): National Academies Press (US); 2016 3, Available from: https://wwwncbinlmnihgov/books/NBK396398/27905704

[2] Plath D (2009). International policy perspectives on Independence in old age. J Aging Soc Policy.

[3] Arrigoitia MF, West K, Peace S (2018). Towards critical intersections of ageing, housing and well-being. Home Cult.

[4] 4430.0 - disability, ageing and Carers, Australia: summary of findings. 2018. Available from: https://www.abs.gov.au/ausstats/abs@.nsf/mf/4430.0.

[5] Afram B, Verbeek H, Bleijlevens MH, Hamers JP (2015). Needs of informal caregivers during transition from home towards institutional care in dementia: a systematic review of qualitative studies. Int Psychogeriatr.

[6] Jacobson J, Gomersall JS, Campbell J, Hughes M (2015). Carers’ experiences when the person for whom they have been caring enters a residential aged care facility permanently: a systematic review. JBI Database System Rev Implement Rep.

[7] Sury L, Burns K, Brodaty H (2013). Moving in: adjustment of people living with dementia going into a nursing home and their families. Int Psychogeriatr.

[8] Schulz R, Belle SH, Czaja SJ, McGinnis KA, Stevens A, Zhang S (2004). Long-term care placement of dementia patients and caregiver health and well-being. JAMA.

[9] Silverstein M, Tur-Sinai A, Lewin-Epstein N (2020). Intergenerational support of older adults by the ‘mature’ Sandwich generation: the relevance of National Policy Regimes. Theor Inq Law.

[10] Australian Institute of Health and Welfare A. Informal Carers 2019. Available from: https://www.aihw.gov.au/reports/australias-welfare/informal-carers. Updated 11 September 2019

[11] Moore KJ, Dow B (2015). Carers continuing to care after residential care placement. Int Psychogeriatr Assoc.

[12] Bramble M, Moyle W, McAllister M (2009). Seeking connection: family care experiences following long-term dementia care placement. J Clin Nurs.

[13] Sussman T, Dupuis S (2012). Supporting a relative’s move into long-term care: starting point shapes family members’ experiences. Can J Aging.

[14] Givens JL, Lopez RP, Mazor KM, Mitchell SL (2012). Sources of stress for family members of nursing home residents with advanced dementia. Alzheimer Dis Assoc Disord.

[15] Hainstock T, Cloutier D, Penning M (2017). From home to ‘home’: mapping the caregiver journey in the transition from home care into residential care. J Aging Stud.

[16] Eika M, Espnes GA, Soderhamn O, Hvalvik S (2014). Experiences faced by next of kin during their older family members’ transition into long-term care in a Norwegian nursing home. J Clin Nurs.

[17] Konietzny C, Kaasalainen S, Dal-Bello Haas V, Merla C, Te A, Di Sante E (2018). Muscled by the system: informal caregivers’ experiences of transitioning an Older adult into long-term care. Can J Aging/La Revue canadienne du vieillissement.

[18] Crawford K, Digby R, Bloomer M, Tan H, Williams A (2015). Transitioning from caregiver to visitor in a long-term care facility: the experience of caregivers of people with dementia. Aging Ment Health.

[19] Martz K (2015). Actions of hospice nurses to alleviate guilt in family caregivers during residential care transitions. J Hosp Palliat Nurs.

[20] Davies S, Nolan M (2006). ‘Making it better’: self-perceived roles of family caregivers of older people living in care homes: a qualitative study. Int J Nurs Stud.

[21] Graneheim UH, Johansson A, Lindgren BM (2014). Family caregivers’ experiences of relinquishing the care of a person with dementia to a nursing home: insights from a meta-ethnographic study. Scand J Caring Sci.

[22] Sirunyan AM, Tumasyan A, Adam W, Ambrogi F, Asilar E, Bergauer T (2018). Elliptic flow of charm and strange hadrons in high-multiplicity p+Pb collisions at sqrt [s_{NN}]=8.16 TeV. Phys Rev Lett.

[23] Davison TE, Camoes-Costa V, Clark A (2019). Adjusting to life in a residential aged care facility: perspectives of people with dementia, family members and facility care staff. J Clin Nurs.

[24] Gaugler JE, Roth DL, Haley WE, Mittelman MS (2008). Can counseling and support reduce burden and depressive symptoms in caregivers of people with Alzheimer's disease during the transition to institutionalization? Results from the new York University caregiver intervention study. J Am Geriatr Soc.

[25] Kokonya A, Fitzsimons V (2018). Transition to long-term care: preparing older adults and their families.(report). MedSurg. Nursing.

[26] Ryan AA, Scullion HF (2000). Nursing home placement: an exploration of the experiences of family carers. J Adv Nurs.

[27] Arksey H, O'Malley L (2005). Scoping studies: towards a methodological framework. Int J Soc Res Methodol.

[28] Munn Z, Peters MDJ, Stern C, Tufanaru C, McArthur A, Aromataris E (2018). Systematic review or scoping review? Guidance for authors when choosing between a systematic or scoping review approach. BMC Med Res Methodol.

[29] Hong QN, Pluye P, Bujold M. et al. Convergent and sequential synthesis designs: implications for conducting and reporting systematic reviews of qualitative and quantitative evidence. Syst Rev. 2017;6:61. 10.1186/s13643-017-0454-2. 10.1186/s13643-017-0454-2 PMC5364694 28335799

[30] Pace R, Pluye P, Bartlett G, Macaulay AC, Salsberg J, Jagosh J (2012). Testing the reliability and efficiency of the pilot mixed methods appraisal tool (MMAT) for systematic mixed studies review. Int J Nurs Stud.

[31] Thomas J, Harden A (2008). Methods for the thematic synthesis of qualitative research in systematic reviews. BMC Med Res Methodol.

[32] Barken R, Lowndes R (2018). Supporting family involvement in long-term residential care: promising practices for relational care. Qual Health Res.

[33] Kallianis V, Joubert L, Gorman S, Posenelli S, Lethborg C (2017). “Unexpected and distressing”: understanding and improving the experience of transferring palliative care inpatients to residential care. J Soc Work End Life Palliat Care.

[34] Kelsey SG, Laditka SB, Laditka JN (2010). Caregiver perspectives on transitions to assisted living and memory care. Am J Alzheimers Dis Other Dement.

[35] Lloyd D (2010). Crisis placement of the elderly in nursing homes: a qualitative study of the lived experience of primary caregivers. Perspectives.

[36] O’Shea F, Weathers E, McCarthy G (2014). Family care experiences in nursing home facilities. Nurs Older People.

[37] Palacios-Cena D, Leon-Perez E, Martinez-Piedrola RM, Cachon-Perez JM, Paras-Bravo P, Velarde-Garcia JF (2019). Female family caregivers’ experiences during nursing home admission: a phenomenological qualitative study. J Gerontol Nurs.

[38] Pearson A, Nay R, Taylor B (2004). Relatives’ experience of nursing home admissions: preliminary study. Australas J Ageing.

[39] Ryan A, McKenna H (2013). Familiarity’ as a key factor influencing rural family carers’ experience of the nursing home placement of an older relative: a qualitative study. BMC Health Serv Res.

[40] Bleijlevens MH, Stolt M, Stephan A, Zabalegui A, Saks K, Sutcliffe C (2015). Changes in caregiver burden and health-related quality of life of informal caregivers of older people with dementia: evidence from the European RightTimePlaceCare prospective cohort study. J Adv Nurs.

[41] Gaugler JE, Mittelman MS, Hepburn K, Newcomer R (2010). Clinically significant changes in burden and depression among dementia caregivers following nursing home admission. BMC Med.

[42] Gaugler JE, Mittelman MS, Hepburn K, Newcomer R (2014). Identifying at-risk dementia caregivers following institutionalization: the nursing home admission-burden and nursing home admission-depression prognostic tools. J Appl Gerontol.

[43] Gaugler JE, Pot AM, Zarit SH (2007). Long-term adaptation to institutionalization in dementia caregivers. Gerontologist.

[44] Metzelthin SF, Verbakel E, Veenstra MY, van Exel J, Ambergen AW, Kempen G (2017). Positive and negative outcomes of informal caregiving at home and in institutionalised long-term care: a cross-sectional study. BMC Geriatr.

[45] McKechnie V, Barker C, Stott J. The effectiveness of an internet support forum for carers of people with dementia: a pre-post cohort study. J Med Internet Res. 2014;16(2). 10.2196/jmir.3166 PMC3961748 24583789

[46] Brodaty H, Donkin M (2009). Family caregivers of people with dementia. Dialogues Clin Neurosci.

[47] Sörensen S, Duberstein P, Gill D, Pinquart M (2006). Dementia care: mental health effects, intervention strategies, and clinical implications. Lancet Neurol.

[48] Noone D, Stott J, Aguirre E, Llanfear K, Spector A. *Meta*-analysis of psychosocial interventions for people with dementia and anxiety or depression. Aging Mental Health. 2019;23(10):1282–91. 10.1080/13607863.2018.1495177. 30328711

