# Supplementary material for: Factors contributing to the mental health outcomes of carers during the transition of their family member to residential aged care: a systematic search and narrative review
Source: BMC Geriatr. 2022 May 17;22:433. doi: 10.1186/s12877-022-03105-4 (PMC9115935; doi:10.1186/s12877-022-03105-4)
Supplement: Supplementary file 1 — Additional file 1. [file 12877_2022_3105_MOESM1_ESM.docx]

Box S1. Full Search Strategy

*PICO*

P family carers of older people

I transition to a residential aged care facility

C before and after admission to Residential Aged Care

O mental health outcomes and quality of life

*Strategy: PubMed and PsycINFO*

1 Aging* OR ageing* OR geriatrics* OR geriatric patients OR elderly* OR older adults OR residents

2 Admission* OR facility admission OR transition*

3 Elder care OR nursing homes OR residential care institutions OR residential care OR residential aged care facility OR long term care OR care home

4 1 AND 2 AND 3

5 Quality of life OR mental health OR mental wellbeing OR wellbeing* OR depression* OR anxiety* OR caregiver burden* OR burden* OR guilt* OR distress* OR stress* OR loss of control OR grief*

6 Family members OR caregivers* OR carer* OR social support OR family* OR family relations OR support* OR support groups OR relativ*

7 5 AND 6

8 4 AND 7

11 Limit to human trials

12 Limit to English language, publication year 2004-2019

*Search Strategy: CINAHL***# Query Limiters/Expanders**

S1

(ageing or elderly or older or adults or seniors) OR aging OR (geriatrics or older adults or elderly or aged or older or elder or elderly) OR geriatric patients OR (older person or older adult) OR (older people or older adults)

S2

family OR (family members or relative or carers or caregivers) OR family members as caregivers OR family members perspectives OR (informal caregivers or family caregivers or informal carers or family carers) OR (social support or family support)

S3

admission OR facility admission OR transition of care OR transition

S4

(elder care or older care or aged care or eldercare) OR (elder care or assisted living or nursing home) OR (residential aged care facility or residential aged care or nursing home or aged care home) OR (residential aged care facility or nursing home or aged care home) OR long term care OR long-term care facilities OR (residential care or nursing home or long term care or care home)

S5

(quality of life or well being or well-being or health-related quality of life) OR mental health OR (depression or anxiety) OR (caregiver burden or caregiver stress or caregiver fatigue or caregiver burnout or caregiver strain) OR guilt OR (loneliness or social isolation) OR (distress or anxiety or stress or psychological or depression)

S6

S1 AND S2 AND S3 AND S4 AND S5

S7

English language, publication year 2004-2019, source type restricted to journals and dissertations
