# Supplementary material for: Factors contributing to the mental health outcomes of carers during the transition of their family member to residential aged care: a systematic search and narrative review
Source: BMC Geriatr. 2022 May 17;22:433. doi: 10.1186/s12877-022-03105-4 (PMC9115935; doi:10.1186/s12877-022-03105-4)
Supplement: Supplementary file 2 — Additional file 2. [file 12877_2022_3105_MOESM2_ESM.docx]

**Box S2. Quality assessment scores using MMAT (Pace et al., 2012).**

1. Qualitative 4. Quantitative descriptive Overall quality

score

| References | 1.1 | 1.2 | 1.3 | 1.4 | 1.5 | 1.6 |  | 4.1 | 4.2 | 4.3 |  |
| --- | --- | --- | --- | --- | --- | --- | --- | --- | --- | --- | --- |
| Barken & Lowndes, 2018, Canada  Abeles and Hafeli, 2014 | Yes | Yes | Yes | Yes | Yes | No |  |  |  |  | *** |
| Bleijlevens et al, 2014, Holland  Abeles and Hafeli, 2014 |  |  |  |  |  |  |  | Yes | Yes | Yes | **** |
| Bramble et al., 2009, Australia  Abeles and Hafeli, 2014 | Yes | Yes | Yes | Yes | Yes | No |  |  |  |  | *** |
| Crawford et al., 2015, Australia  Abeles and Hafeli, 2014 | Yes | Yes | Yes | Yes | Yes | No |  |  |  |  | *** |
| Davies & Nolan, 2006, UK  Abeles and Hafeli, 2014 | Yes | Yes | Yes | Yes | Yes | No |  |  |  |  | *** |
| Davison et al., 2019, Australia  Abeles and Hafeli, 2014 | Yes | Yes | Yes | Yes | Yes | No |  |  |  |  | *** |
| Eika et al., 2014, Norway  Abeles and Hafeli, 2014 | Yes | Yes | Yes | Yes | Yes | No |  |  |  |  | *** |
| Gaugler et al., 2007, USA  Abeles and Hafeli, 2014 |  |  |  |  |  |  |  | Yes | Yes | Yes | **** |
| Gaugler et al., 2010, USA  Abeles and Hafeli, 2014 |  |  |  |  |  |  |  | Yes | Yes | Yes | **** |
| Gaugler et al., 2014, USA  Abeles and Hafeli, 2014 |  |  |  |  |  |  |  | Yes | Yes | Yes | **** |
| Givens et al, 2012, USA  Abeles and Hafeli, 2014 | Yes | Yes | Yes | Yes | Yes | No |  |  |  |  | *** |
| Hainstock et al., 2017, Canada  Abeles and Hafeli, 2014 | Yes | Yes | Yes | Yes | Yes | No |  |  |  |  | *** |
| Kallianis et al., 2017, Australia  Abeles and Hafeli, 2014 | Yes | Yes | Yes | Yes | Yes | No |  |  |  |  | *** |
| Kelsey et al., 2010, USA  Abeles and Hafeli, 2014 | Yes | Yes | Yes | Yes | Yes | No |  |  |  |  | *** |
| Konietzny et al., 2018, Canada | Yes | Yes | Yes | Yes | Yes | No |  |  |  |  | *** |
| Lloyd, 2010, Canada | Yes | Yes | Yes | Yes | Yes | No |  |  |  |  | *** |
| Metzelthin et al., 2017, Holland |  |  |  |  |  |  |  | Yes | Yes | Yes | **** |
| O'shea et al., 2014, Ireland | Yes | Yes | Yes | Yes | Yes | No |  |  |  |  | *** |
| Palacios-Ceña et al., 2019, Spain | Yes | Yes | Yes | Yes | Yes | No |  |  |  |  | *** |
| Pearson et al., 2004, Australia | Yes | Yes | Yes | Yes | Yes | No |  |  |  |  | *** |
| Ryan & McKenna, 2013, Ireland | Yes | Yes | Yes | Yes | Yes | No |  |  |  |  | *** |
| Schulz et al., 2004, USA |  |  |  |  |  |  |  | Yes | Yes | Yes | **** |
| Sussman & Dupuis, 2012, Canada | Yes | Yes | Yes | Yes | Yes | No |  |  |  |  | *** |
| *meets 25% of MMAT criteria.  **meets 50% of MMAT criteria.  ***meets 75% of MMAT criteria.  ****meets 100% of MMAT criteria. | 1.1 Qualitative objective or question  1.2 Appropriate qualitative approach or method  1.3 Description of the context  1.4 Description of participants and sampling  1.5 Description of data collection and analysis  1.6 Discussion of researchers’ reflexivity | | | | | | | 4.1 Appropriate sampling and sample  4.2 Justification of measurement (valid/standard)  4.3 Acceptable response rate | | | |
